# The Predictive Role of Biomarkers for Leprosy Prophylaxis in Contacts of Patients Who Are Indices of the Disease: A Systematic Review of the Literature

**DOI:** 10.1155/mi/6163972

**Published:** 2026-02-17

**Authors:** Luiza Raquel Tapajós Figueira, Marcos Jessé Abrahão Silva, Lucas Vinicius Moraes da Silva, Rebecca Lobato Marinho, Keitty Anne Silva Neves, Thiago Augusto Ferreira dos Anjos, Lilian Cristina Santos Sinfronio da Silva, Daniele Melo Sardinha, Everaldina Cordeiro dos Santos, Luana Nepomuceno Gondim Costa Lima

**Affiliations:** ^1^ Postgraduate Program in Parasitic Biology in the Amazon (PPGBPA), University of Pará State (UEPA), Belém, Pará, Brazil; ^2^ Postgraduate Program in Epidemiology and Health Surveillance (PPGEVS), Evandro Chagas Institute (IEC), Ananindeua, Pará, Brazil, iec.pa.gov.br; ^3^ Bacteriology and Mycology Section (SABMI), Evandro Chagas Institute (IEC), Ananindeua, Pará, Brazil, iec.pa.gov.br

**Keywords:** biomarkers, household contact, inflammatory, leprosy, subclinical infection

## Abstract

Leprosy continues to be an important public health problem, particularly in endemic regions such as Brazil, India, and Indonesia. Household contacts of multibacillary (MB) patients represent a high‐risk group for subclinical infection due to prolonged exposure and high bacillary load. Host biomarkers have emerged as promising tools for identifying early infections and guiding prophylactic interventions. This systematic review aimed to identify and synthesize evidence on inflammatory and immune biomarkers associated with susceptibility to leprosy and disease progression among contacts of index cases, evaluating their potential predictive and diagnostic value. The study followed the Preferred Reporting Items for Systematic Reviews (PRISMA) 2020 guidelines and was registered in the International Prospective Register of Systematic Reviews (PROSPERO) (CRD420251111469). We searched CAPES, SciELO, PubMed, ScienceDirect, EMBASE, Scopus, and EBSCO databases for original studies published between 2012 and 2025, with no language restrictions. Two review authors independently selected studies using the Rayyan software, and methodological quality was assessed using the ROBIS tool. The biomarkers most frequently investigated in the studies were particularly tumor necrosis factor‐alpha (TNF‐α) and interleukin (IL)‐10, which play regulatory roles in the host. Elevated levels of TNF‐α, interferon‐γ (IFN‐γ), IL‐6, and IL‐4 were associated with a higher risk of subclinical infection among contacts of MB patients, indicating a polyfunctional immune profile. On the other hand, paucibacillary (PB) contacts exhibited lower cytokine activation, suggesting partial protection. Additional promising markers included anti‐Mce1A, PGL‐I IgM, and CCL4, detected primarily by enzyme‐linked immunosorbent assay (ELISA) and polymerase chain reaction (PCR) methods. In summary, inflammatory and immune biomarkers—especially TNF‐α, IL‐10, IFN‐γ, and anti‐Mce1A—demonstrate potential as predictive indicators of subclinical leprosy infection. Their combined use may increase risk stratification and allow early therapeutic intervention in endemic settings. However, longitudinal validation studies are required prior to clinical application.

## 1. Introduction

Leprosy is a neglected tropical disease (NTD**)** [1], caused by *Mycobacterium leprae* and, more recently, *M. lepromatose*, discovered in 2008. It is operationally classified as paucibacillary (PB) when the patient has up to five skin lesions and a negative smear test (examination to detect the bacillus) and multibacillary (MB) when the patient has more than five skin lesions and/or positive bacilloscopy for the bacillus, considered the advanced form of the disease [2–4].

As well as its clinical forms, indeterminate, the first phase, and tuberculoid, where lesions and loss of sensitivity are already observed, both PB, already in the MB form, are borderline leprosy with various complications with more symmetrical distribution of plaques or nodules, and the more severe Virchowian form (lepromatous) with numerous diffuse lesions, extensive bacterial infiltration in the skin and nerves, with deficient cellular immunity and exacerbated humoral response [2, 5].

It is a growing threat to global health, with a high concentration in Asia, South America, and Africa, and a higher prevalence in some specific countries (India, Brazil, and Indonesia) with about 80% of reported cases [1, 2]. In the Americas, Brazil led with a large majority of cases (92%), followed by a smaller number in countries such as Venezuela, Colombia, and Paraguay. It is the only American country that has failed to achieve the WHO leprosy control target of less than one case per 10,000 inhabitants, with a detection rate of 1.32 cases, ranking second in incidence [6, 7].

Incidence rates are influenced by socioeconomic, environmental, and public health logistics factors, showing that leprosy still represents an epidemiological challenge, especially in tropical and underdeveloped countries, with Brazil being an important global focus due to its high prevalence and high specific regional rates [8]. Despite the long incubation period of leprosy, it is worth noting that, for infection to occur, an extended period of exposure to the bacteria is necessary, and even then, most cases remain asymptomatic [9, 10].

In this context, it is worth highlighting the intradomestic contacts (people who resided, lived, or lived with the patient in the home environment in the 5 years prior to diagnosis of the disease) of patients with leprosy, who, due to their routine proximity, are more likely to become ill than the general population, especially if the patient has the MB form, due to the higher bacilli load, increasing the potential transmission and, over time, the contact organism changes according to the evolution of the infection, and may present a latent infection [11–14].

Depending on these conditions, in addition to the environmental and biological conditions of the host, especially those with an immune response mediated predominantly by the humoral pathway (Th2) and activation of regulatory T lymphocytes (Treg), where there is no efficiency to control the disease, fierce responses will occur, developing in MB forms, evolving into Type 1 leprosy reactions (reverse reaction) and Type 2 (erythema nodosum) reactions that, without timely treatment, result in tissue and nerve damage, permanent disabilities, reduced mobility of the extremities, and even blindness [2, 15, 16].

Understanding the mechanisms that regulate this response may help prevent and treat the most severe forms of the disease. Host biomarkers may be useful as a means of detection in patient serum and may be applicable for estimating risk [17] and the biological characteristics of *M. leprae* contribute to a lesser extent to clinical manifestations [15]. The use of host markers is a useful complementary approach for improving the predictive accuracy of early infection diagnosis in contacts of index patients, enabling the selection of the appropriate therapeutic approach for adequate treatment and reducing the likelihood of errors and adverse effects [18–22].

Thus, this study sought to review the correlation between the immunological characteristics presented in the household contacts of leprosy patients and the different presentations of their biomarkers as tools to aid in diagnosis and/or prognosis to monitor the possible evolution of the disease and, thus, use a prophylactic approach to prevent the development of the disease and subsequent transmission. The objective is to systematically search the literature for biomarkers that are potentially more prevalent in contacts of leprosy patients as tools to guide more appropriate therapeutic and prognostic approaches for these patients.

## 2. Materials and Methods

### 2.1. Study Design

This is a systematic review conducted in accordance with the Preferred Reporting Items for Systematic Reviews (PRISMA) 2020 [23], with the protocol under code CRD420251111469 registered on the platform International Prospective Register of Systematic Reviews (PROSPERO).

### 2.2. Search Strategy and Eligibility Criteria

To construct the search strategy and research question, we used the “PICO” mnemonic, recommended in evidence‐based practice (EBP), which proposes that clinical problems that arise in healthcare, teaching, or research practice be broken down according to an organization, as shown in Table 1.

**Table 1:** Distribution of the PICO strategy for eligibility of studies.

P: Population	Contacts of people with leprosy
I: Interventions	Detection of specific biomarkers (serological, molecular, and genetic).
C: Comparison	Healthy people, healthy and unhealthy contacts of leprosy patients.
O: Outcome	Predictive biomarkers for the development of active or subclinical diseases.

The following research question was defined: What are the potential biomarkers that could be used as predictors to aid in the early detection, monitoring, and/or screening of contacts of patients with leprosy?

Studies were eligible for inclusion if they presented a description of household, peridomestic, and social contacts of leprosy; risk and/or protective factors for healthy contacts; observational, cross‐sectional, case–control, or cohort studies evaluating laboratory tests involving leprosy biomarkers, such as immune and inflammatory biomarkers. Publications in all languages were included, with no restrictions on data or geographical area, between 2012 and 2025.

Studies classified as previous reviews, case reports, interviews, letters to the editor, experimental or ecological studies that were only descriptive without analysis of the results, as well as those that only mentioned tests without referring to the biomarker analyzed, were excluded, as were protein biomarkers because they are specific to each individual. The searches were conducted in July 2025 in the following databases: Periódicos CAPES; SciELO; PubMed; ScienceDirect, EMBASE, Scopus, and EBSCO. Controlled words from Medical Subject Headings (MeSH) and Boolean operators were used to locate relevant content. A combination of controlled and uncontrolled descriptors was used, as indicated by each database, which were “Leprosy,” “biomarkers,” “Patient contact,” and “family.”

### 2.3. Study Selection and Data Extraction

Duplicate studies were excluded in the Zotero reference manager, and articles were selected using the Rayyan application, which is used in systematic reviews to facilitate the article selection process. The reading took place in three phases: in the first, searches were performed in the databases; in the second, two authors read the title and abstract of the articles in order to separate them for the next phase; and finally, in the third stage, the articles were read in full to select those that met the pre‐inclusion criteria established for the final analysis. A third reviewer was consulted in case of doubts or disagreements.

### 2.4. Risk of Bias and Applicability

**Figure 1:**
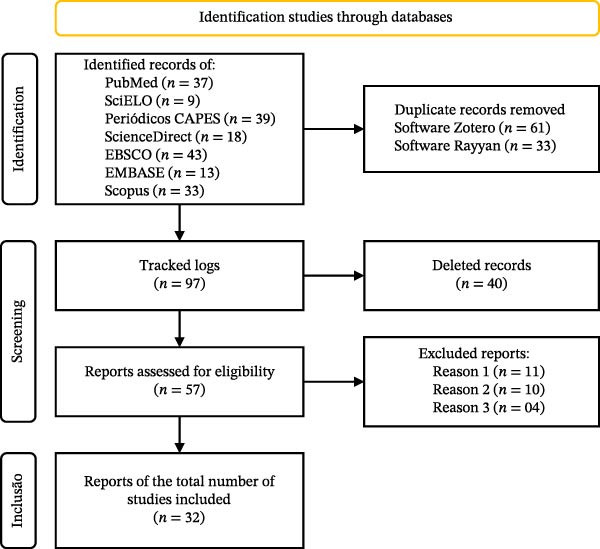
Selection process of eligible studies to compose the systematic review.

Methodological quality was assessed using the ROBINS‐I Tool [24]. ROBINS‐I examines seven domains of risk of bias (confounding, participant selection, exposure classification, intervention deviations, missing data, outcome measurement, and selection of reported results), classifying each study as low, moderate, high, or critical risk. Two independent assessors performed the assessment, and disagreements were resolved by a third investigator. The ratings were synthesized in a summary plot generated by the risk of bias visualization tool (ROBVIS), and the overall risk of bias for each study was assigned according to the highest risk identified in any domain. The results are presented in Figure 1, generated by the ROBVIS [25].

## 3. Results

**Figure 2:**
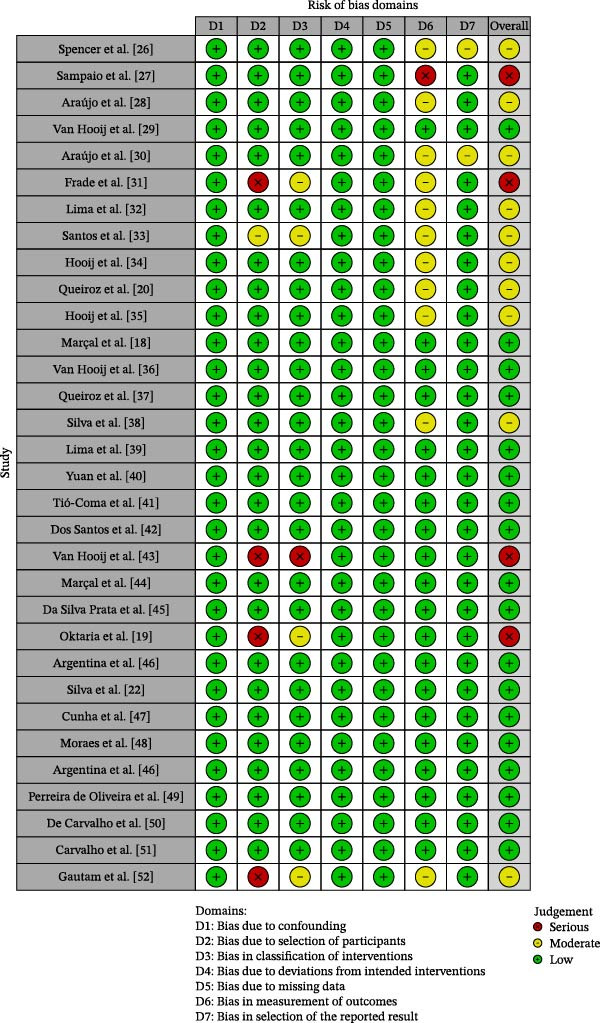
Summary plot of bias risk assessment.

The search resulted in 191 articles selected from PubMed (36 articles), SciELO (9 articles), Periódicos CAPES (39 articles), Science Direct (18 articles), EBSCO (43 articles), EMBASE (13 articles), and Scopus (33 articles) during the initial search. After removing duplicates, 97 articles were evaluated, and 40 articles were removed after screening titles and abstracts. A total of 57 articles underwent a full‐text review. Of these, 11 were excluded because they did not correspond to the objective, 10 articles were excluded because they had other focuses, and four were removed because they did not present feasible results. Thus, 32 articles were included in the final qualitative analysis. The detailed screening process and the reasons for article exclusion are summarized in Figure 2.

### 3.1. Study Characteristics

Of the 32 articles included in this study, they ranged from 2012 to 2025, with the years with the highest number of studies being 2021, with seven articles and 2023 with four articles, which were mostly cohort studies (*n* = 17) followed by cross‐sectional studies (*n* = 11). The most prevalent country was Brazil with 20 studies, followed by Bangladesh with five studies. The sample sizes of the participants in these articles ranged from 27 to 5352. The most frequent detection method used was the Enzyme‐Linked Immunosorbent Assay (ELISA), followed by the polymerase chain reaction (PCR), which was sometimes complementary, adding to the specificity and sensitivity in detecting the target. Table 2 provides a detailed overview of the study characteristics.

**Table 2:** Characteristics of the selected articles related to biomarkers.

Year of the Author	Country	Sample	Detection method	Classification	Biomarker	Study design
Spencer et al. [26]	Philippines	69	WBA and ELISA	Immune	LID‐1 and ML2028	Cohort
Sampaio et al. [27]	Brazil	30	WBA and ELISA	Inflammatory	IFNγ, IL‐4 and IL‐5	Cohort
Araújo et al. [28]	Brazil	1352	ELISA serology, anti‐PGL‐I, and PCR	Immune	PGL‐I	Transverse
Van Hooij et al. [29]	Bangladesh	1110	WBA, ELISA, and UCP‐LFA	Inflammatory	CCL4	Exploratory
Araújo et al. [30]	Brazil	104	ELISA, anti‐PGL‐I, and PCR	Immune	PGL‐I	Cohort
Frade et al. [31]	Brazil	93	Quick test, ELISA, and NDO‐LID	Immune	PGL‐I, ND‐O‐TAMPA, and TAMPA‐I	Cohort
Lima et al. [32]	Brazil	89	ELISA	Immune	anti‐Mce1A	Cohort
Santos et al. [33]	Brazil	35	QPCR of blood, qPCR skin smear, and qPCR skin biopsy	Immune	PGL‐I	Cohort
Hooij et al. [34]	Brazil (510), China (220), and Ethiopia (77)	807	WBA	Immune	PGL‐I	Cohort
Queiroz et al. [20]	Brazil	27	CBA and ELISA	Immune	MFI in lymphocytes T CD4+CD25+, and CD8+CD25+	Transverse
Hooij et al. [35]	Bangladesh	184	WBA and ELISA	Inflammatory	ApoA1, IL‐1Ra, S100A12, CCL4, PCR, IL‐10, IP‐10, and αPGL‐I	Cohort
Marçal et al. [18]	Brazil	49	Culture	Inflammatory	IFN‐γ and IL‐10	Transverse
Van Hooij et al. [36]	Bangladesh	300	PCR, RLEP, and Qpcr	Inflammatory and immune	S100A12 and CCL4/CRP	Cohort
Queiroz et al. [37]	Brazil	188	CBA	Inflammatory	IFN‐γ and CCL2	Longitudinal
Silva et al. [38]	Brazil	77	ELISA	Immune	NDO‐HSA	Cohort
Lima et al. [39]	Brazil	82	PCR, RLEP, and ELISA	Immune	anti‐Mce1A	Cohort
Yuan et al. [40]	China	98	RT‐qPCR	Inflammatory	IL‐8, CCL2, and SERP	Cohort
Tió‐Coma et al. [41]	Bangladesh	5352	RT‐qPCR	Inflammatory	RISK4LEP	Cohort
Dos Santos et al. [42]	Brazil	106	Extraction and genotyping of DNA	Inflammatory	TGF β	Transverse
Van Hooij et al. [43]	Bangladesh	50	WBA and ELISA	Inflammatory	IL‐6, CCL4, and TNF‐α	Cohort
Marçal et al. [44]	Brazil	160	Culture and ELISA	Inflammatory	TNF, IL‐10, IL‐4, and/or TGF‐β	Transverse
Da Silva Prata et al. [45]	Brazil	260	PCR, RLEP, and ELISA	Inflammatory	HO‐1	Cohort
Oktaria et al. [19]	Indonesia	401	Multiplex immunoassay kit with conjugated magnetic spheres	Inflammatory	IL‐6, IL‐8, and IL‐10	Case–control
Argentina et al. [46]	Indonesia	54	RT‐qPCR	Inflammatory	HBD‐3 and cathelicidin	Transverse
Silva et al. [22]	Brazil	34	Liquid chromatography–mass spectrometry	Immune	PGL‐I	Transverse
Cunha et al. [47]	Brazil	37	Culture and cytometric bead array	Inflammatory	IL‐6, TNF, NFI‐γ, and IL‐17; CXCL8, CCL2, CXCL9, and CXCL10	Transverse
Moraes et al. [48]	Brazil	512	ELISA	Inflammatory	PTX3	Transverse
Argentina et al. [46]	Indonesia	54	ELISA	Inflammatory	Cathelicidin	Transverse
Perreira de Oliveira et al. [49]	Brazil	257	Culture and sphere‐based immunoassays	Inflammatory	TNF and IFN‐γ	Transverse
De Carvalho et al. [50]	Brazil	71	Multiplex microspheres and EIA	Inflammatory	IL‐1β, TNF, IFN‐γ, IL‐1Ra, IL‐9, PDGF, G‐CSF, and IL‐2; CXCL8, CCL3, CCL4, CCL2, and CCL5	Observational
Carvalho et al. [51]	Brazil	75	Microsphere immunoassay	Inflammatory	CXCL8, CCL3, CCL4, and CXCL10; IL‐1β, TNF‐α, IFN‐γ, IL1Ra, and IL‐9, PDGF, G‐CSF, and IL‐2	Cohort
Gautam et al. [52]	India	40	Bradford method: liquid chromatography–mass spectrometry and in‐silico characterization	Inflammatory	AIBG and Hp 1	Cohort

### 3.2. Main Conclusions of the Study

The systematic review included 32 articles published between 2012 and 2025, mostly cohort and cross‐sectional studies, conducted mainly in Brazil and India. The most frequently investigated biomarkers were pro‐ and anti‐inflammatory cytokines, especially tumor necrosis factor‐alpha (TNF‐α), cited in seven studies [18, 20, 27, 47, 49–51] and IL‐10, present in four [18, 19, 35, 44].

The frequency of these molecules demonstrates that their expression plays a fundamental role in the dynamics between immune protection and disease progression. While TNF‐α is associated with the activation of the cellular response against *Mycobacterium leprae*, IL‐10 is related to regulation, counterbalancing the immune response, which may favor the persistence of the bacillus [18, 35].

It was observed that the contacts of patients (MB) had immune profiles similar to those of the index cases themselves [26, 47, 49]. These individuals had elevated levels of TNF‐α, interferon‐γ (IFN‐γ), IL‐10, IL‐6, and IL‐4, constituting a polyfunctional profile that suggests greater susceptibility to subclinical infection [50, 51]. In contrast, contacts with patients (PB) exhibited less activation of these pathways, which may indicate a lower risk of progression to active disease [37]. This immunological proximity between contacts and MB patients reinforces the idea that high bacillary load and exposure time are decisive factors for the progression of infection, indicating that there was a distinction between PB and MB contacts.

Noting that the MB form exhibits marked secretion of antigen‐specific cytokines, suggesting a higher risk of developing active disease, contacts of PB patients had a less intense immune response, which may indicate relative protection against the clinical progression of leprosy [37, 47]. This difference in immune profile suggests that the classification of the index patient should be considered in the risk stratification of contacts [18, 26, 27, 34, 36, 53].

Regarding immune markers, it was observed that the expression of serum IgM PGL‐1 (Phenolic Glycolipid I) titer is specific to *M. leprae* and tends to progress over months, depending on the type of patient (PB or MB) [22, 26, 34], being more expressed in MB and minimal in PB. Other antibody levels related to adaptive immunity are similar between contacts and PB patients, such as NDO‐HSA, LID‐1, and NDO‐LID, indicative of a limited response to the bacteria. On the other hand, the dysregulation of such levels causes a possible activation of the disease [36, 38].

Promising studies are looking for ways to monitor potential seropositivity in contacts with patients, such as anti‐Mce1A, which has great potential for serological biomarkers in the diagnosis of leprosy and screening of household contacts of index patients, contributing to early identification or subclinical cases through immune status, using the low‐cost and noninvasive ELISA laboratory method [32, 39].

As well as CCL4 levels, a chemokine involved in the host’s immune response to *Mycobacterium leprae* infection, whose increased levels can exacerbate the immune response, and C‐reactive protein (CRP), which is produced by the liver in the acute phase and is predictive of leprosy reactions and treatment evaluation, both of which aid in understanding the patient’s immune status and can contribute to early diagnosis and proper management of leprosy, and prevention of serious complications [36, 54].

Since it is difficult to predict the onset of the disease. In addition, as most tests aim to detect adaptive immune responses to *M. leprae* or its antigens, these methods can only evaluate cases where there has been prolonged exposure to the bacillus, which often detects mainly cases of MB or cases where the disease is already at a more established stage.

## 4. Discussion

The articles included in the qualitative synthesis process of this systematic review evaluated the potential contribution of cytokines as possible biomarkers in the diagnostic support of leprosy to assist in the immediate initiation of treatment for the effective control of leprosy in household contacts [18–20, 22]. The study of inflammatory molecules present in leprosy contacts is fundamental to understanding the immune mechanisms underlying both infection control and the development and variation in the clinical presentations of the disease [55].

Studies affirm the importance of integrating serological, molecular, genetic, and inflammatory biomarkers to improve early diagnosis and monitoring of leprosy [56]. Inflammatory biomarkers, including mast cell degranulation and increased expression of annexin A1, are related to Type 1 and 2 leprosy immune reactions and can aid in the identification and management of these complications [33]. The presence of antibodies against the Mce1A protein of *Mycobacterium leprae* is a differential serological marker, in which IgA antibodies indicate contact with the bacillus, IgM reflects active disease, and IgG is associated with follow‐up after treatment [28].

In addition, genetic polymorphisms in genes such as NOD2 and IFN‐γ susceptibility and immune response to leprosy demonstrate a relevant role of genetic factors in pathogenesis [57]. Lipidomic analyses of the skin by mass spectrometry have identified characteristic lipid profiles that distinguish infected individuals, suggesting a noninvasive and accurate approach to diagnosis [58].

When individuals come into contact with *M. leprae* or *M. lepromatosis*, the immune system is activated by the interaction of the bacillus with cells of the innate system, mainly macrophages and dendritic cells, which recognize the microorganism through pattern recognition receptors, such as Toll‐like receptors (TLRs). The activation of these receptors results in the production of pro‐inflammatory cytokines, such as IL‐6 and IL‐8, which participate in cell recruitment and activation of the initial inflammatory response, constituting an essential mechanism to contain bacterial proliferation in the early stages [59].

As it is a chronic inflammatory disease, it is associated with its host through its immune response, and the levels and types of cytokines imply susceptibility to clinical severity and immune reactions that may occur during the course of the disease [60]. In other words, the clinical spectrum depends on the type of immune response, mainly the production of cytokines, whether they are pro‐inflammatory related to the effective cellular immune response or anti‐inflammatory associated with a Th2‐type response, which favors more severe forms of the disease [13, 61].

Therefore, the pathogenesis of leprosy, is articulated through macrophages that mediate interactions between the host and *M. leprae*. They are classified as M1 (pro‐inflammatory), which secrete, for example, IFN‐γ and TNF‐α, and M2 (anti‐inflammatory), which are related to tissue repair along with fibrosis and are activated by interleukin (IL)‐4, IL‐10, IL‐13, and TGF‐β, F [17]. Cytokines, on the other hand, are the link between innate and adaptive immunity. Within their family, there are ILs that are produced by macrophages, indicating the direction for lymphocytes that secrete IFN‐γ, TNF‐α, which together modulate the host’s ability to respond to pathology and other humoral activities [62].

In the disease, the balance (or imbalance) in the polarization of M1/M2 macrophages determines the clinical form of the disease, the degree of control of the bacillus, and the pathological consequences. When there is a dominant M1 response, the host is able to control the infection, limiting bacterial multiplication and the extent of lesions. With M2 predominance, there is a higher bacillary load, extensive lesions, immune escape, and increased transmission to contacts. In addition, the tissue microenvironment, cytokines (especially IFN‐γ for M1 and IL‐4/IL‐10 for M2), and pathogen characteristics directly influence macrophage polarization and, therefore, the evolution of infection [63].

The prognosis of *M. leprae* infection among close contacts of leprosy patients is influenced by multiple risk factors, including individual metabolic profile, nutritional status, cohabitation, and residence in endemic areas. This occurs because these individuals have increased levels of specific cytokines such as pro‐inflammatory IL‐6, anti‐inflammatory IL‐10, pro‐inflammatory IL‐8, and anti‐inflammatory IL‐10 [19]. The authors argue that the immunological similarities shared by contacts and patients living in the same environment indicate that cohabitation may influence the outcome of the immune response and express a pattern of cytokines and chemokines associated with an elevated risk [20].

In this sense, in leprosy, elevated levels of IL‐6 are associated with general inflammation, being more present in unstable forms, where they influence tissue remodeling, and less pronounced in more resistant forms, such as tuberculoid. IL‐8 functions as a chemokine recruiting neutrophils to local inflammation, aiding in defense, although it is not abundant in the Virchowian form, where the inflammatory response is reduced. In contrast, IL‐10 is crucial in the Virchowian form, where it induces immunosuppression and inhibits the effective cellular response (macrophages and T lymphocytes), allowing the uncontrolled proliferation of *M. leprae* and progression to the MB [64–66].

Studies suggest that their findings provide immunological characteristics of patient contacts and may be useful tools for diagnosis and/or prognosis, serving as a way to monitor possible subclinical infections [18–20, 47], especially when exposed to the MB form, since susceptibility is attributed to the patient’s higher bacillary load index, exposure time, and the clinical profile of patients in each endemic area [19, 42, 47].

The high frequency of TNF‐α and IL‐10 in these cases demonstrates the importance of inflammatory responses both in the initial containment of *M. leprae* and in promoting progression to severe forms of the disease. The identification of multifunctional profiles in contacts of MB patients reinforces the hypothesis that proximity and intensity of exposure increase the probability of subclinical infection and subsequent disease, justifying expanded surveillance of these groups [67].

On the other hand, there is no consensus on which of the cytokines presented cause susceptibility or protection against disease, as they are subject to other factors such as metabolic status and lipid mediators [19, 42]. The interaction of these inflammatory molecules in contact shows a delicate balance between protective immunity and immunosuppression, which influences the clinical spectrum of leprosy, ranging from the tuberculoid form, due to cellular inflammatory response and bacillary control, to the Virchowian form of immunosuppression and high bacillary load [64]. This immune profile shared between index cases and their contacts corroborates that the host response plays a central role in maintaining the chain of transmission [68].

Among the antibodies studied, anti‐PGL‐I is the most traditional and widely used in epidemiological and functional value in contact monitoring. PGL‐I (phenolic glycolipid I) is a species‐specific antigen of *Mycobacterium leprae* with great importance for the diagnosis and prognosis of leprosy. Serology for anti‐PGL‐I antibodies is used as a marker of bacterial load, with higher positivity in patients with MB forms of the disease, although it can also be detected in PB forms [69].

Studies show that contacts who are seropositive for anti‐PGL‐I antibodies have a higher risk of developing leprosy, estimated to be about seven times higher compared to seronegative contacts [56]. In addition, the risk of developing MB forms among these seropositive contacts can be up to 24 times higher. These data suggest that serological testing for PGL‐I may serve as a prognostic marker in identifying contacts with a higher probability of becoming ill, allowing for early intervention and surveillance [70, 71].

Recent studies focus on the *M. leprae* Mce1A protein, an adhesion mechanism that aids in bacillus invasion and is useful in screening the immune response to anti‐Mce1A antibodies. It has proven effective in distinguishing between patients with active disease and those already treated, surpassing the anti‐PGL‐I biomarker in sensitivity and specificity, as the latter is more sensitive in MB cases, which limits its general use [32, 53, 70]. Thus, anti‐Mce1A contributes to early identification, especially in the detection of subclinical cases, and is a valuable tool for signaling immune status and indicating latent progression, overcoming some limitations of PGL‐I, particularly in its predictive levels for early cases [39].

Another relevant class includes anti‐LID‐1 and anti‐ND‐O‐LID antibodies, which combine bacillus antigens to increase the sensitivity and specificity of serological tests. Recent evidence has shown that the detection of antibodies against these antigens is associated with more reactive forms of leprosy and can predict the risk of leprosy reaction episodes, especially Type II forms, which are related to complications and sequelae [72, 73].

There was also methodological diversity in the included studies, with ELISA and PCR being the most frequent methods for biomarker detection. These approaches were often used in a complementary manner, suggesting that methodological integration increases diagnostic accuracy and the robustness of findings. This combination reinforces the feasibility of future use of standardized biomarker panels for cost‐effective contact tracing in endemic areas, aligning diagnostic needs with the economic context of high‐risk groups and optimizing Hansen’s bacillus control [74, 75].

In terms of operational advantages, rapid serological tests, such as MLflow (which detects antibodies against PGL‐I), offer benefits compared to complex laboratory methods, such as cytokine assays. MLflow is a rapid, practical, and easy‐to‐perform test in the field that does not require sophisticated laboratory equipment. With sensitivity and specificity similar to traditional ELISA, it speeds up diagnosis and large‐scale monitoring, making it ideal for use by healthcare professionals in basic units and remote areas with limited resources [76, 77].

The exclusive use of cytokine markers in the prognosis of leprosy contacts faces challenges that limit its effective application, primarily the dependence on sophisticated laboratory infrastructure, specific equipment, and trained technicians, factors that restrict implementation in primary care settings, especially in geographic areas with limited resources [78, 79]. Additionally, high costs prevent its large‐scale use for population screening. Biological and temporal variability in cytokine expression can also result in nonspecific data, making it difficult to clearly interpret the immune status of the contact, especially in the early stages of infection. Finally, prolonged response time delays timely intervention in people at risk [80].

Considering these limitations, the integration of rapid serological tests and cytokine analysis emerges as a more robust alternative for screening contacts with leprosy. This interdisciplinary strategy proposes that rapid serological tests, such as MLflow for the detection of antibodies against PGL‐I, be used initially as screening tools in the field [81]. These tests offer speed, ease of use, and low cost, and are suitable for implementation in basic health units and remote communities. Contacts identified as seropositive can then undergo complementary assessments, which involve detailed analysis of the cytokine profile, in order to better understand the immune response and monitor the possible progression of the disease [82].

Furthermore, the integrated approach provides for periodic monitoring of contacts using both techniques, allowing for continuous surveillance that adapts to individual risk and local prevalence. Technical training and partial decentralization of cytokine testing to regional reference laboratories will optimize resources, improve geographic coverage, and ensure diagnostic quality. Thus, the combined use of these methods offers greater sensitivity and specificity in the early identification of cases and in assessing the risk of developing leprosy, facilitating more assertive clinical interventions and contributing to the effective control of the disease [83].

Furthermore, the set of clinical/epidemiological factors, as well as serological and molecular data, must be taken into account for an integrated approach, especially in cases of PB patients, since the main means of detection is anti‐PGL‐I antibodies, which are bacillary markers characteristic of high bacillary load, that is, MB [70]. Molecular methods such as PCR are feasible through detection using clinical samples (nasal swab, skin biopsy, and blood) complement serology, identifying the direct presence of *M. leprae*, as well as ELISA, which signals immune risk. In an integrated manner, they can intervene early to control transmission [84].

Through this systematic review of the literature, gaps were observed in the literature on the detailed use of serum biomarkers to predict whether the disease will develop, since they depend on numerous factors in addition to the type of exposure that arises. Although there is consistent evidence on the predictive role of certain cytokines, the field still lacks longitudinal studies to validate their use as an early screening tool. In addition, integrated strategies that combine immune biomarkers with clinical, epidemiological, and molecular data may represent preventive monitoring in leprosy in the future [85].

The heterogeneity of findings among different endemic regions suggests the influence of social, nutritional, and environmental determinants on the expression of biomarkers, reinforcing the need for multicenter validation before incorporating these markers into clinical protocols. Although some studies have proposed combinations of cytokines as promising diagnostic panels, there is still no consensus on which biomarkers offer the best sensitivity and specificity for predicting subclinical infection [19].

The findings underscore the urgency of investing in public policies that incorporate biomarker‐based testing into contact tracing in endemic areas. Systematic screening of household contacts and continuous immune monitoring can significantly reduce the time between infection and diagnosis, allowing for early interventions. The use of these biomarkers can support targeted prophylactic strategies, reducing transmission and the impact of leprosy in vulnerable populations [86, 87].

## 5. Conclusions

The results of this systematic review reinforce the promising role of inflammatory and immune biomarkers as predictive and monitoring tools for leprosy, especially among contacts of MB patients. Cytokines such as TNF‐α, IL‐10, IFN‐γ, and IL‐6 stood out for their high frequency and association with susceptibility to subclinical infection, while serological markers such as anti‐PGL‐I and anti‐Mce1A demonstrated potential in screening exposed contacts, allowing early identification of individuals at risk of developing the disease. The body of evidence demonstrates a dynamic balance between protective response and immunosuppression, which determines the clinical evolution of *Mycobacterium leprae* infection.

The combination of serological markers and inflammatory cytokines can improve diagnostic accuracy and guide more precise prophylactic and therapeutic intervention strategies. The integrated use of rapid tests, such as MLflow, with complementary laboratory analyses (ELISA, PCR) is a cost‐effective approach, especially in endemic regions with limited laboratory resources. This integration represents a significant advance in epidemiological surveillance and leprosy control, allowing monitoring to be targeted at higher‐risk groups based on objective immune parameters.

## Funding

No funding was received for this manuscript.

## Conflicts of Interest

The authors declare no conflicts of interest.

## Data Availability

The data that support the findings of this study are available from the corresponding author upon reasonable request.
